# The COVID-19 Response in Nebraska: How Students Answered the Call

**DOI:** 10.5888/pcd17.200269

**Published:** 2020-08-13

**Authors:** Sabrine Chengane, Anlan Cheney, Sierra Garth, Sharon Medcalf

**Affiliations:** 1 College of Public Health, University of Nebraska Medical Center, Omaha, Nebraska; 2 Department of Epidemiology, College of Public Health, University of Nebraska Medical Center, Omaha, Nebraska

## Abstract

The Student Response Team at the University of Nebraska Medical Center answered the statewide call to assist local health departments during the coronavirus disease 2019 (COVID-19) pandemic. As a voluntary student-led effort, the SRT assisted health departments to conduct contact tracing, monitor social media, and educate the public. Their experience demonstrates how students can increase the public health surge capacity of local health departments while gaining applied experience during public health emergencies. This call-to-action commentary proposes that SRTs should be formed, trained, and deployed through academic institutions across the nation and the globe, during and beyond the current pandemic.

**Summary**

**What is already known on this topic?**

Emory University and The University of North Carolina were 2 of the first schools of public health with student response teams using the expertise of graduate students in the control and containment of outbreaks.

**What is added by this report?**

The University of Nebraska Medical Center (UNMC) College of Public Health established a Student Response Team (SRT) in 2015 that expanded upon these models and positioned the SRT to assist Nebraska’s coronavirus disease 2019 (COVID-19) response.

**What are the implications for public health practice?**

The UNMC SRT demonstrates how students can increase the public health surge capacity of local health departments while gaining applied experience during public health emergencies.

## Introduction

Emory University (1) and the University of North Carolina (2) were among the first schools of public health that created programs to use the expertise of graduate students in the control and containment of outbreaks (3). In 2002, Emory University created the Student Outbreak and Response Team (SORT) to provide students with hands-on experience in emergency response through a collaboration with the Centers for Disease Control and Prevention (3). Team Epi-Aid was created in 2003 at the University of North Carolina School of Public Health, presenting its students with opportunities to support local and state health departments and gain practical skills (3).

In the spring of 2015, the co-director (S.M.) of the Center for Biosecurity, Biopreparedness, and Emerging Infectious Diseases established the University of Nebraska Medical Center (UNMC) Student Response Team (SRT). She believed that public health students were equipped to assist in more areas than traditional teams had ventured into, so the UNMC SRT was created for 3 specific scenarios: 1) an epidemiology outbreak team to assist local health departments with outbreak investigation and control (eg, conduct case and contact interviews, data entry), 2) a points-of-dispensing (POD) assistance team to assist public health emergency response coordinators with mass dispensing or immunization clinics (eg, serve as greeters, screeners, dispensers, immunizers), and 3) a digital response team to assist volunteer agencies in systematic monitoring of social media (eg, conduct data mining, data verification, and geomapping) (3). Local health departments could rely on an increased workforce to augment their epidemiology staff in an outbreak and their emergency preparedness staff with POD operations either in an actual mass dispensing or immunization event or for annual exercises (trainings very close to a real-life event, typically conducted by public agencies). A global volunteer group, the Standby Task Force, partnered with the UNMC SRT in 2017 to offer training and volunteer opportunities in actual disasters across the world to monitor social media and to geomap distress calls and other information.

The coronavirus disease 2019 (COVID-19) pandemic in 2020 would provide the most extensive response involvement that UNMC SRT had experienced to date. In the 2019–2020 school year, 33 public health students joined the SRT as members, and 17 members volunteered to assist health departments with the COVID-19 response. Additionally, 20 public health students who were not members of the SRT answered the call for volunteers. We present here some background information on this SRT and its activities related to COVID-19 in Nebraska communities.

## Recruitment, Governance, and Training

The SRT recruits students from the College of Public Health (3), including those in the certificate, master’s, and doctoral programs. New students in these programs initially learn about the SRT at the orientation before the fall semester begins. Officers and other members of the SRT give a presentation to students, highlighting trainings that take place during the year and potential volunteer opportunities. The SRT also recruits students at the Student Involvement Fair during the first week of the fall semester (3). In recent years, most of the recruited students were enrolled in the Master of Public Health (MPH) program, typically with a concentration in health promotion or epidemiology. Regular member meetings, which usually have around 20 students in attendance, provide platforms for informing members, training for deployments, and recruiting new members. If the SRT needs to activate at any time, current members are notified first, because they have received essential information during training sessions (3).

SRT activities are possible with the support of the SRT’s faculty advisor (S.M.) and its collaboration with members of the executive board. Each year, students are nominated for positions on the executive board: president, vice president, secretary, and treasurer. Together, the executive board organizes training opportunities and coordinates potential deployments. The board also promotes regular member meetings through fliers, emails, and announcements to their classmates during in-person class sessions. Member meetings are used as training sessions for the team. At these meetings, the executive board assists the health departments and other guest speakers with the trainings. The executive board also meets monthly to plan for trainings. A training is planned for each semester to prepare volunteers for immediate deployment to assist health departments in case of an emergency (Table 1).

**Table 1:** Student Response Team Training and Deployments, University of Nebraska Medical Center, 2020

Area of Focus	Competencies/Skills	Training Frequency	Historical Deployments
Epidemiology outbreak	Assisting health departments with Contact tracing Symptoms monitoring (Research Electronic Data Capture [REDCap]; Vanderbilt University) Social media monitoring	One-hour training, once a semester	2020 COVID-19 pandemic response
Points of dispensing	Assist with mass dispensing or immunization clinics	One-hour training, once a semester	Mass testing of students for latent tuberculosis in 2016 after a positive case was identified at a local school
Digital response	Systematic monitoring of social media during natural disasters (eg, hurricanes): Data mining Data verification Geo-mapping	One-hour training, once a semester	Assisted with monitoring social media for distress calls in the Houston area during Hurricane Harvey in 2017

Trainings are implemented to prepare SRT volunteers to assist health departments with an outbreak investigation, POD assistance, and digital response. Although the topics of these trainings may vary, depending on the needs of the community, the goal is to provide students with information needed for immediate deployment. In epidemiology outbreak training, a guest speaker, usually a member of a local health department, explains fundamental epidemiologic concepts and demonstrates how to apply these concepts in the field. Multiple health departments also conduct POD training for students. The interactive simulation teaches students how to assist with the mass dispensing of medications or vaccinations during a public health emergency. This training is especially important because local health departments would work with the SRT and other health professionals during an emergency event to ensure that the correct medical countermeasures are given to the community in a timely manner. The digital response training involves acquainting students with the process of accessing, data mining, and coding various social media platforms, particularly Twitter and Facebook, to find information pertaining to a specific public health emergency. In the past, the SRT partnered with the Standby Task Force to monitor and analyze tweets during hurricanes in the Gulf of Mexico. The ultimate goal of aggregating the data was to enable local responders to help those in affected areas. Recently, the SRT monitored social media to better understand the public’s perception of COVID-19.

## Historical Deployments

SRTs at UNMC have deployed in epidemiology outbreak teams, POD teams, and digital response teams (3). The experiences with POD operations have been the most frequent. Several surrounding county health departments have enlisted the students to assist with annual full-scale exercises, not as health personnel but in greeter, screener, dispenser, and educator operational roles. Students were able to experience an actual modified POD operation in 2016, when it was used to implement mass testing of hundreds of students for latent tuberculosis after a positive case was identified at a local school.

In 2017, the SRT was activated by the Standby Task Force to assist with monitoring social media for distress calls in the Houston area during Hurricane Harvey. Messages were geomapped by providing latitude and longitude coordinates for the originating location or address and were uploaded to a document shared with the Standby Task Force. Records for calls and associated location points were shared with the US Coast Guard and provided valuable data for rescue operations. From 1,000 miles away, SRT members worked together as a group and individually, contributing lifesaving information to responders on the ground. More recent teams were able to offer similar assistance during Hurricane Dorian.

## COVID-19 Deployments

In February 2020, SRT members discussed the idea of monitoring social media to capture sentiments and opinions of Nebraskans about the imported cases of COVID-19 to Camp Ashland and the UNMC quarantine unit. As events evolved and the city of Omaha reported community-transmitted cases, the SRT took the initiative to collect information biweekly on Facebook and Twitter. The SRT used 2 keywords in data collection: *Nebraska* and *coronavirus.* The data that were collected informed the SRT and health department partners of the nature and quality of information that circulated in the community. Thus, it helped health departments in Nebraska address misinformation and rumors and give the population accurate health advice and guidance.

On March 10, 2020, the SRT received a call for volunteers from the Douglas County (Omaha) Health Department (DCHD) to support contact tracing, symptom tracking, test result notification, and social media monitoring. The volunteers were trained to assist the epidemiology team immediately. Volunteers were provided with information about the current epidemic situation in the city of Omaha and the state of Nebraska and received instructions on the different activities that they could join. The volunteers made telephone calls to inquire about the health status of people who came in contact with confirmed coronavirus cases and provided instructions on how to self-quarantine. Volunteers used Research Electronic Data Capture (REDCap) (Vanderbilt University) for symptom monitoring and EpiInfo software (Centers for Disease Control and Prevention) to enter patient information and COVID-19 test results, providing a valuable opportunity to gain technical skills in public health software applications.

Following the work that SRT volunteers accomplished with DCHD, the SRT received calls for volunteers from other health departments across the state in March and April 2020 (Table 2). In addition to activities described above, volunteers participated in public education and outreach about chronic disease and COVID-19. During contact tracing, volunteers provided quarantine recommendations to COVID-19 patients and fielded questions and offered advice about managing comorbidities such as diabetes, asthma, and high blood pressure. Some volunteers counselled people with confirmed cases about staying current with their medications and ensuring that their supplies would last through their isolation period. Some volunteers also helped to address concerns of the population through managing health department social media accounts by replying to messages and comments that the departments received.

**Table 2:** Health Department Requests for Student Response Team Assistance During Coronavirus Disease 2019 (COVID-19) Response, Nebraska, 2020

Requesting Organization	Type of Assistance Requested	Number of Volunteers^a^
Health department 1	Social media monitoring and content editing	4
Health department 2	Data entry into Research Electronic Data Capture (REDCap) (Vanderbilt University) Telephone assistance and routing	3
Health department 3	Telephone follow-up on exposures	5
Health department 4	Data entry into a simple screening tool Telephone coverage	6
Health department 5	Active monitoring of travelers and positive confirmed cases Daily (2 times) check-in for symptoms for any traveler or positive confirmed cases	2
Health department 6	Contact tracing and potential telephone triage Patient monitoring	2
Health department 7	Social media monitoring and replies Data entry and analysis Education material development	9
Health department 8	Contact tracing	10
Health department 9	Contact tracing Social media monitoring and dissemination	3
Health department 10	Contact tracing and telephone follow-up on exposure	8
Health department 11	Remote assistance	1

a Some volunteers may have volunteered with more than 1 health department.

The SRT’s role in public education and outreach also included identifying and supporting the most vulnerable and underserved populations in their communities during the COVID-19 response. Bilingual volunteers helped translate education materials, create infographics, and liaise with communities speaking Arabic, Kurdish, and Spanish, in rural and urban parts of the state. Some volunteers worked to generate reports on the outbreak magnitude among meatpacking plant workers. Outreach included a presentation to an audience of high school students by an SRT volunteer and the team’s faculty advisor about COVID-19 and the SRT’s role in the response. At the peak of activities, 34 students were deployed to 11 health departments across the state.

## Student Perspectives

A feedback assessment survey was deployed (by A.C.) to 50 individuals listed in the SRT COVID-19 volunteer registry. Fifteen volunteers responded (30%). The survey asked volunteers to list their degree type and concentration area and to respond in open text boxes about why they were attracted to the SRT, which work with the team resonated with them the most, how they assisted the COVID-19 response, how SRT volunteer experiences affected them academically and professionally, and whether they engaged any aspects of chronic disease management during the COVID-19 response. Volunteers were also invited to offer suggestions for how to integrate chronic disease management in emergency-oriented work and how students at other institutions could pursue similar opportunities. Text inputs were analyzed by coding responses to compare commonalities within the following categories: attraction, resonance, academic impact, professional impact, and chronic disease management.

Of the 15 volunteers responding, 9 studied for an MPH degree, 3 for a doctoral degree, and 2 for a certificate in public health. One volunteer was a medical student. Nearly half of the volunteers studied epidemiology, and one-third studied health promotion. The remaining respondents studied health services research and administration, general public health, or medicine. All of the survey participants volunteered during the COVID-19 response.

Students were attracted to volunteer with the SRT for 4 main reasons: to contribute to their communities, to gain specific pandemic experience, to participate in trainings, and to gain professional exposure. In the spirit of volunteerism, they were “attracted to the idea of helping the community in a time of need.” Those motivated specifically by the opportunity to “contribute to the pandemic” also cited the attraction to “get immediately involved.” While many volunteers responded to the SRT’s call specific to COVID-19, some joined the SRT earlier after attending trainings such as POD for public health emergencies. The experiences offered through the SRT, volunteers emphasized, are a “complement to the area of public health I wanted to work in.”

Volunteers also said they found resonance in the community-based and practice-based aspects of SRT opportunities. Some said engaging in social media monitoring helped them “feel more prepared to address the COVID-19 sentiments among friends and family and within the community.” Another volunteer, noting how “public health is always present but only acknowledged during health crisis,” gained “an added level [of] gratitude and appreciation for public health workers who had to mobilize and organize education efforts in rural Nebraska.” Several volunteers were fulfilled in assisting multilingual or refugee communities, some of which they belong to themselves. A volunteer serving a Latino community affected by chronic diseases, including cancer, diabetes, cardiovascular disease, and obesity, noted that many of its members were not aware they were considered to be high risk. “As a member of this community I felt that it was my responsibility to inform, educate, and provide accurate information,” the volunteer said. Volunteers also articulated an understanding that health departments, many of which are underfunded and underresourced, were strained during the COVID-19 response. As one volunteer explained, “Despite having the training and skills to help, I felt impotent in the face of the early chaos of our response to the pandemic. I hoped I could lend my time to worthwhile efforts to contain the devastation by volunteering with the Student Response Team.”

In terms of academic impact, epidemiology student volunteers recalled how the response aligned with coursework in outbreak investigations and allowed them to observe “[a] team go through all the investigation steps that we weren’t actively participating in ourselves.” Where their opportunities to participate in real-time data collection had previously been limited, volunteers were afforded experience in this aspect of the epidemiologic response and through social media monitoring. One volunteer, whose experience inspired them to take a SAS (SAS Institute Inc) programming class as an elective, said the exposure “added to my understanding of the need for data collection and for innovation in determining how and what to collect.” Other volunteers learned how to use REDCap and EpiInfo for the first time. For one medical student, medical knowledge provided advantages in educating people about their illness while new insights gained from contact tracing “will aide my clinical skills.”

Volunteers felt the experience was rewarding in terms of professional development as well, through working with multidisciplinary teams and being exposed to public health practice. In their varied experiences integrating into teams that were already functioning at health departments across the state, volunteers practiced how to collaborate within an organization firsthand. In many cases, this work was conducted remotely. With this early professional exposure to remote working environments, volunteers felt the ability to communicate and coordinate with senior staff and organizations regardless of location was greatly strengthened. Volunteers also celebrated how they learned “the art-part of public health” in making clear and acceptable communications in uncertain times. As a contact tracer, a volunteer shared how “it was a new experience for me to be a calm, knowledgeable authority to strangers that were nervous and unsure. I think it helped me grow as a leader and a communicator and will stay with me throughout my career.” Working directly with health departments also created opportunities and connections for career development. Some volunteers aim to one day work for local health departments, and others graduated during the response and were employed as contact tracers at the departments where they volunteered. For graduating students facing challenges with job availability and hiring freezes, volunteering with the SRT “helped bridge the gap between being a student and finding full-time work in public health.”

## Public Health Implications

The UNMC SRT is a student-led initiative that seeks to expand the capacity of local public health departments and provide students with opportunities to gain applied public health experience in emergencies. During Nebraska’s COVID-19 response, the UNMC SRT provided opportunities for applied practice experience and professional development of the future public health workforce. Furthermore, activities increased recognition of the public health role and value among other health professions and community engagement in health emergency response. The SRT calls all students and universities to consider establishing response teams and offers the following recommendations and insights:

- **SRTs provide applied practice opportunities for the future public health workforce (** **2** **)**. As a student-led effort, SRTs encourage autonomy and empower creativity of future health professionals. Serving on an SRT is a unique opportunity for students to apply the knowledge acquired in academic settings and gain field experience that facilitates employability after graduation. The implementation and training of SRTs prepare students for deployment in case of public health emergencies, regardless of their educational background. For this approach to be successful, it is crucial to ensure the availability of public health leadership and quality training of the health workforce in local communities. SRTs are an accelerated learning platform for public health programs and one that future employers — health departments, public and private institutes, foundations, and others — can support through partnerships with degree-granting institutions.
- **SRTs promote visibility of public health leadership in health emergencies.** SRTs can expand resources and networking opportunities for students. Because students with any background can be trained to perform in key areas, SRTs provide a platform for interprofessional collaboration. Collaborating with different health professions like medicine, pharmacy, nursing, and others promotes interprofessional teamwork and knowledge exchange.
- **SRTs can focus on communities in response efforts**. During COVID-19, having direct contact with community members through telephone calls presented an opportunity for the UNMC SRT to help health departments better serve rural and marginalized populations. This contact also facilitated the dissemination of health information in various languages, given that some volunteers are multilingual or are willing to serve in their home communities. After volunteering with communities that were more severely affected by the challenges of COVID-19 and chronic disease in tandem, some volunteers raised concerns about addressing social determinants of health and health disparities at the early stages of emergency response. In the future, SRTs could undertake more long-term and preventive postures in lieu of their response activities. Accordingly, community ties should be maintained beyond emergencies, and their perspectives should be the focus when planning training and response activities. For example, training sessions could include risk communications specific to managing chronic disease and comorbidities in emergencies.
- **Strategies to improve and sustain SRTs**. Following the example set by North Carolina’s Epi-Aid (2), SRTs are recommended to evaluate their efforts across the breadth of recruitment, training, and volunteer activities. When not responding to a public health emergency, SRT members can participate in planning and evaluation efforts that provide yet another opportunity for students to practice foundational public health competencies.

The UNMC SRT provides early evidence on how students can increase the public health personnel resources of local health departments, particularly during periods considered a national emergency. This commentary provides insight into ways to prepare public health students, integrate students into the workflow of health departments, and evaluate the contributions of students in achieving overall program goals. Lesson learned from the UNMC SRT experience can serve as a foundation that other academic institutions can adopt to meet the needs of both their students and collaborating health departments.
